# Disseminated *Acanthamoeba* Infection with Necrotic Skin Lesions and Granulomatous Vasculitis, United States

**DOI:** 10.3201/eid3204.251201

**Published:** 2026-04

**Authors:** Maria Koshy, Carrie Flynn, Marat Kribis, Jennifer McNiff, Matthew Grant, Shana Elizabeth Gleeson

**Affiliations:** Yale School of Medicine, New Haven, Connecticut, USA (M. Koshy, M. Kribis, J. McNiff, M. Grant, S.E. Gleeson); Brigham and Women’s Hospital, Boston, Massachusetts, USA (C. Flynn).

**Keywords:** *Acanthamoeba*, nitroxoline, granulomatous vasculitis, necrotic skin lesions, dupilumab, free-living amoeba, parasitic infection, parasites, immunosuppressed patients, Florida, United States

## Abstract

An elderly man, on dupilumab therapy for asthma and sinus polyposis, sought care for necrotic skin lesions. Biopsy revealed granulomatous vasculitis and he received immunosuppressive therapy but worsened. We diagnosed an *Acanthamoeba* infection that was treated with multidrug therapy including nitroxoline. Clinicians should be aware of this rare etiology for infectious vasculitis.

*Acanthamoeba* spp. are free-living amoeba (FLA) that can be pathogenic in humans. *Acanthamoeba* amebae are most known for causing contact lens-related keratitis. Nonkeratitis invasive disease is primarily an opportunistic infection, and often fatal (*1*,*2*).

The number of persons at risk for opportunistic infections is increasing and is compounded by a growing number of drugs with unique targets on the inflammatory cascade (*3*). Dupilumab is a fully human monoclonal antibody that inhibits interleukin-4 and interleukin-13 cytokines and the T2 inflammatory response (*4*). It was first approved in 2017 for the treatment of atopic dermatitis and has since been used to treat several allergic diseases (*4*).

We report an elderly man on dupilumab therapy who sought care for progressive necrotic skin lesions and granulomatous vasculitis. We describe the clinical manifestations, diagnostic approach, and management considerations in this patient who was ultimately diagnosed with disseminated *Acanthamoeba* infection.

## The Case

A 78-year-old man was admitted to a tertiary-care academic hospital with a 6-month history of progressive skin lesions. The lesions developed after he returned from Florida, USA. The lesions began as reddish nodules that progressed to develop central darkening. Some lesions evolved into deep ulcers; others developed necrosis with eschar. The lesions began on the legs but progressed to involve all extremities, the trunk, head, and neck. The back was spared. He denied fever but reported a 16-pound weight loss.

His past medical history included asthma and sinus polyposis treated with dupilumab for the past 18 months. He performed nasal rinses with normal saline. He spent winters in Florida, and during his last trip he had exposure to red tide and knee-deep brackish water when engaged in cleaning efforts after a hurricane.

Prior to admission, the patient underwent multiple skin biopsies that revealed focal granulomatous medium-vessel vasculitis with neutrophilic infiltrates, and negative acid-fast bacillus, fungal, and periodic acid–Schiff stains. Clinicians were concerned that the vasculitis was immune-mediated, and he received immunosuppressive therapy including prednisone at doses up to 80 mg daily, mycophenolate mofetil, rituximab, and cyclophosphamide. However, the lesions progressed. He was transferred to our hospital for clinical deterioration requiring a higher level of care.

**Figure 1:**
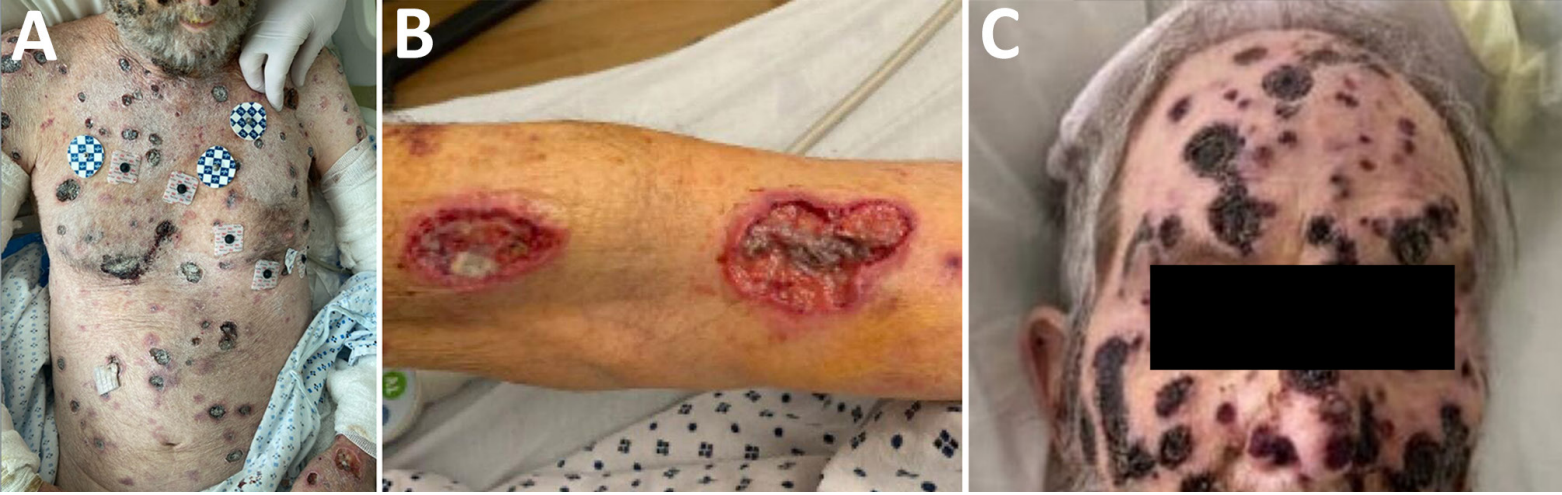
Images of skin lesions on a patient with disseminated *Acanthamoeba* infection with necrotic skin lesions and granulomatous vasculitis. A) Lesions on the patient’s body. B) Deeply ulcerated lesions on the patient. C) Lesions that extended to the head and face.

At physical examination, he was febrile (temperature 102°F), tachycardic (heart rate 122 beats per minute), and had unremarkable blood pressure (130/80 mm Hg). He was frail and ill-appearing. Skin examination revealed extensive lesions in varying stages, some were erythematous and nodular, but the more predominant appearance was that of necrotic lesions with black central eschars (Figure 1 panel A); some lesions had formed deep ulcers (Figure 1 panel B). Head and neck examination disclosed similar lesions (Figure 1 panel C), palatal fistula, and a lesion causing partial loss of left upper eyelid with corneal exposure. Neurologic exam revealed confusion and drowsiness, with no neck stiffness. The remainder of the examination was unremarkable.

**Figure 2:**
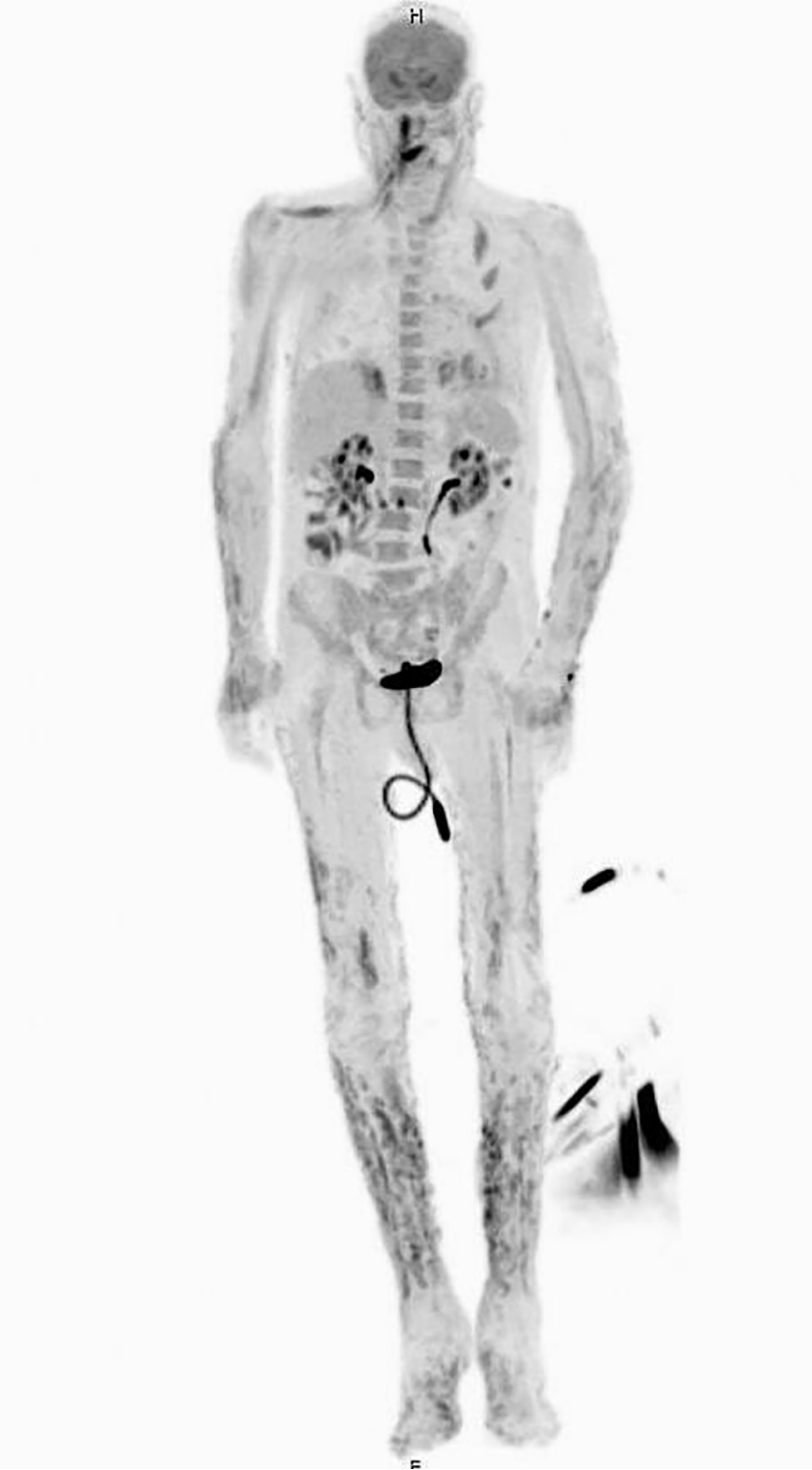
Positron emission tomography/computed tomography showing innumerable widespread ^18^F-fluorodeoxyglucose avid cutaneous and subcutaneous lesions that developed in a patient with disseminated *Acanthamoeba* infection.

The patient’s laboratory results revealed a leukocyte count of 26 × 10^3^ cells/µL (reference range 4–11 × 10^3^ cells/µL), hemoglobin of 8.8 g/dL (reference range 13.2–17.1 g/dL), albumin of 2.1 g/dL (reference range 3.6–5.1 g/dL), and high sensitivity C-reactive protein of 173 mg/L (reference range <1 mg/L). Testing for hepatitis B, C, and HIV was negative. No growth was reported from blood culture. Antinuclear antibodies and antineutrophilic cytoplasmic antibodies were not detected. Complement levels were unremarkable. A positron emission tomography computed tomography revealed innumerable widespread ^18^F-fluorodeoxyglucose avid cutaneous and subcutaneous lesions (Figure 2).

**Figure 3:**
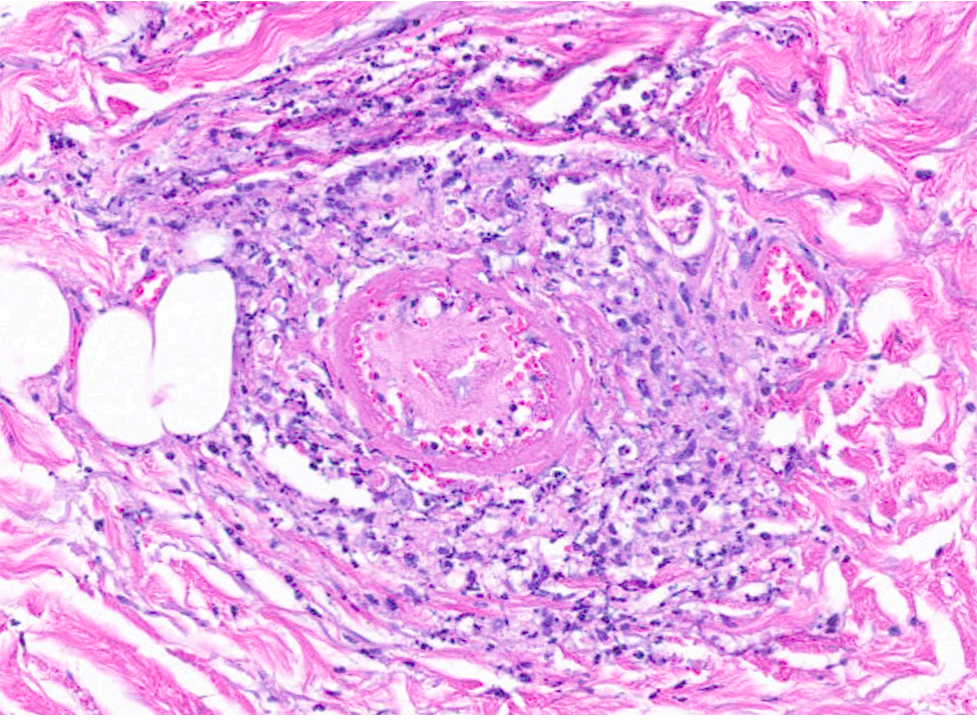
Periodic acid–Schiff positive histiocytoid cells from a patient with disseminated *Acanthamoeba* infection with necrotic skin lesions and granulomatous vasculitis. Hematoxylin and eosin stain; original magnification ×200.

**Figure 4:**
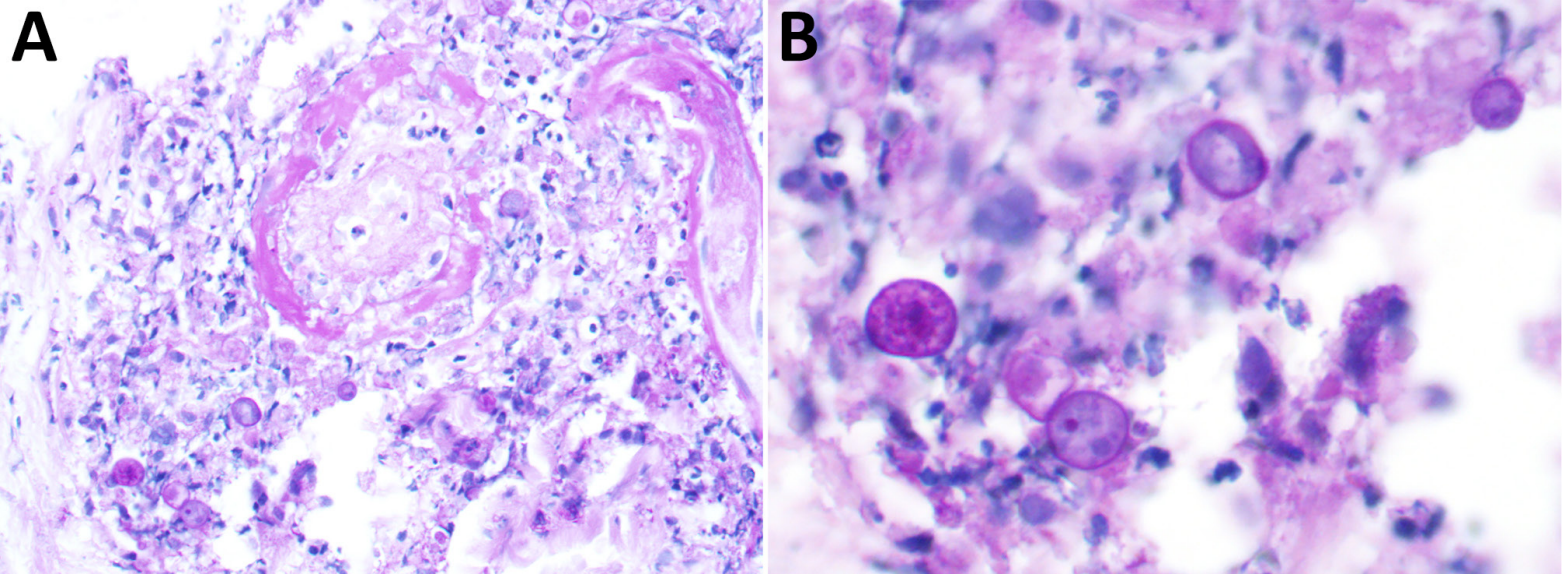
Positive histiocytoid cells from a patient with disseminated *Acanthamoeba* infection with necrotic skin lesions and granulomatous vasculitis. Cells are near the vessel with potential amoebic trophozoites. A) Original magnification ×400; B) Original magnification ×600.

Because of the disease progression despite treatment for immune-mediated vasculitis, the skin biopsy was repeated. The biopsy again showed medium-vessel vasculitis with cutaneous necrosis. On periodic acid–Schiff stain, a few large histiocytoid cells around the vessels stained positive, indicating possible amoebic trophozoites (Figures 3, 4). The cells were confirmed to be *Acanthamoeba* spp. on 18S rDNA amoeba PCR testing. Blood cell–free DNA (Karius, https://kariusdx.com) testing detected high levels of *Acanthamoeba lugdunensis* (400,000 DNA molecules/μL, analytical range of 10–316,000 DNA molecules/μL). Brain magnetic resonance imaging revealed a punctate focus within the right cerebellar hemisphere but no leptomeningeal enhancement. We sent cerebrospinal fluid for free-living amoeba PCR testing to the Centers for Disease Control and Prevention (Atlanta, Georgia, USA). The result was negative, with the comment that although cerebrospinal fluid was an ideal specimen for the detection of *Naegeleria fowleri*, it was not an ideal specimen for *Acanthamoeba* spp.

We started the patient on a multidrug regimen that included sulfadiazine, flucytosine, fluconazole, pentamidine, and miltefosine (*5*). We tapered and then discontinued steroids. The patient continued to deteriorate. On the basis of a separate clinical case of the successful use of nitroxoline in the treatment of *Balamuthia mandrillaris* encephalitis, and unpublished in vitro data from our institution, we obtained Food and Drug Administration approval to use nitroxoline through single-patient expanded-access of an investigational new drug. We initiated the use of nitroxoline 20 days after diagnosis (250 mg 3×/d). Chlorhexidine soaks, anidulafungin (100 mg 1×/d), and isuvaconazole (372 mg 1×/d) were added (*6*,*7*).

After those modifications in treatment, the patient showed initial signs of improvement: some lesions over the face and trunk improved and no new lesions appeared. His fevers briefly resolved. Unfortunately, his renal function worsened and we were concerned that nitroxoline was contributing to kidney injury. We stopped the drug after 7 days, along with pentamidine and sulfadiazine. Many of his wounds showed signs of secondary infection and multiorgan failure developed. The patient died 6 weeks after *Acanthamoeba* infection diagnosis.

## Conclusions

*Acanthamoeba* are ubiquitous in soil and water, and one study from the United States noted their presence in 51% of household water samples (*1*,*2*,*8*). However, invasive disease in humans is rare, and manifestations include granulomatous amoebic encephalitis, cutaneous disease, rhinosinusitis, pulmonary disease, and osteomyelitis. Reports of invasive *Acanthamoeba* infection with HIV cases have increased (*2*). Other risk factors include malignancy and receipt of solid organ or hematopoietic stem-cell transplant (*9*). Tap water nasal irrigation is another well described risk factor and could have played a role in this patient’s infection (*10*).

The treatment of *Acanthamoeba* infections is on the basis of survivor case reports and in vitro drug studies. The Centers for Disease Control and Prevention recommends a multidrug regimen including miltefosine, sulfadiazine, pentamidine, flucytosine, and a nonfluconazole triazole. A host of empiric therapies have been tried (*5*). Nitroxoline is a hydroxyquinolone used to treat urinary tract infections, and has good clinical efficacy and a good safety profile (*11*). Nitroxoline has successfully been used in treating *Balamuthia* encephalitis, and in vitro data from our institution also demonstrated its efficacy in killing *Acanthamoeba castellanii* trophozoites (*6*,^7^). Although this patient ultimately died from the infection, he showed initial signs of improvement, the first during his long illness, after starting nitroxoline.

Increased risk for parasitic infections have been reported with therapies targeting IgE and the T2 inflammatory response. A recent study suggested that although the numbers are low, the disproportionate safety signal warranted investigation (*12*). In the VOYAGE trial, which studied the use of dupilumab in children with asthma, 6 cases of helminthiasis were reported, including enterobiasis and ascariasis (*13*). Although dupilumab is not classically considered an immunosuppressive agent, it possibly increases the risk for parasitic infections.

In summary, we believe the disseminated *Acanthamoeba* infection in our patient occurred because of multiple factors that included his environmental exposure, his age-related immune decline, and dupilumab therapy. FLA are rarely included in reviews on etiology of granulomatous vasculitis. Physicians should be aware of this under-recognized etiology, and this case adds to the body of literature supporting the use of nitroxoline in FLA infections.
